# Serial tissue Doppler imaging in the evaluation of bronchopulmonary dysplasia-associated pulmonary hypertension among extremely preterm infants: a prospective observational study

**DOI:** 10.3389/fped.2024.1349175

**Published:** 2024-04-05

**Authors:** Krishna Revanna Gopagondanahalli, Abdul Alim Abdul Haium, Shrenik Jitendrakumar Vora, Sreekanthan Sundararaghavan, Wei Di Ng, Tze Liang Jonathan Choo, Wai Lin Ang, Nur Qaiyimah Binte Mohamad Taib, Nishanthi Han Ying Wijedasa, Victor Samuel Rajadurai, Kee Thai Yeo, Teng Hong Tan

**Affiliations:** ^1^ Department of Neonatology, KK Women’s and Children’s Hospital, Singapore, Singapore; ^2^ Yong Loo Ling School of Medicine, Singapore, Singapore; ^3^ Lee Kong Chian School of Medicine, Singapore, Singapore; ^4^ Duke—NUS Medical School, Singapore, Singapore; ^5^ Department of Paediatric Cardiology, KK Women’s and Children’s Hospital, Singapore, Singapore

**Keywords:** bronchopulmonary dysplasia, pulmonary hypertension, tissue Doppler imaging, extreme prematurity, conventional echocardiogram

## Abstract

**Objectives:**

To evaluate serial tissue Doppler cardiac imaging (TDI) in the evolution of bronchopulmonary dysplasia-associated pulmonary hypertension (BPD-PH) among extremely preterm infants.

**Design:**

Prospective observational study.

**Setting:**

Single-center, tertiary-level neonatal intensive care unit.

**Patients:**

Infant born <28 weeks gestation.

**Main outcome measures:**

Utility of TDI in the early diagnosis and prediction of BPD-PH and optimal timing for screening of BPD-PH.

**Results:**

A total of 79 infants were included. Of them, 17 (23%) had BPD-PH. The mean gestational age was 25.9 ± 1.1 weeks, and mean birth weight was 830 ± 174 g. The BPD-PH group had a high incidence of hemodynamically significant patent ductus arteriosus (83% vs. 56%, *p* < 0.018), longer oxygen days (96.16 ± 68.09 vs. 59.35 ± 52.1, *p* < 0.008), and prolonged hospital stay (133.8 ± 45.9 vs. 106.5 ± 37.9 days, *p* < 0.005). The left ventricular eccentricity index (0.99 ± 0.1 vs. 1.1 ± 0.7, *p* < 0.01) and the ratio of acceleration time to right ventricular ejection time showed a statistically significant trend from 33 weeks (0.24 ± 0.05 vs. 0.28 ± 0.05, *p* < 0.05). At 33 weeks, the BPD-PH group showed prolonged isovolumetric contraction time (27.84 ± 5.5 vs. 22.77 ± 4, *p* < 0.001), prolonged isovolumetric relaxation time (40.3 ± 7.1 vs. 34.9 ± 5.3, *p* < 0.003), and abnormal myocardial performance index (0.39 ± 0.05 vs. 0.32 ± 0.03, *p* < 0.001). These differences persisted at 36 weeks after conceptional gestational age.

**Conclusions:**

TDI parameters are sensitive in the early evolution of BPD-PH. Diagnostic accuracy can be increased by combining the TDI parameters with conventional echocardiographic parameters. BPD-PH can be recognizable as early as 33–34 weeks of gestation.

## Introduction

Bronchopulmonary dysplasia (BPD), which is currently defined by continued oxygen/respiratory support beyond 36 weeks of gestation, continues to be the most common respiratory morbidity in surviving preterm infants (1). The development of pulmonary hypertension (PH) among infants with BPD often complicates the respiratory course in the vulnerable population. The complex interaction of abnormal vascular remodeling, arrested vascular growth, reduced vascular surface, immature repair mechanisms, reduced expression of pro-angiogenic factors, or over-expression of anti-angiogenic factors have all been linked to abnormal pulmonary vascular resistance in patients with BPD (2–4).

The incidence of BPD-PH in the premature population is approximately 17%–24% but can vary between 27% and 40% with gestation less than 25 weeks and other associated co-morbidities (5–7). There is increased mortality when BPD is complicated by PH (5, 8). The true burden of PH in BPD is unknown due to the poor sensitivity of conventional echocardiographic parameters.

The gold standard for measuring pulmonary arterial pressure is cardiac catheterization; however, this is not practical in the preterm population due to the inherent high risk of invasive nature. Two-dimensional echocardiography is most widely used as an indirect measurement of pulmonary artery pressure and diagnosis of pulmonary hypertension. Although the individual conventional parameters lack sensitivity, the combination of multiple echocardiographic parameters is thought to increase the sensitivity of diagnosis of BPD-PH (9). The proposed timeline for screening PH in BPD is 36 weeks in high-risk infants, but there is limited information on serial echocardiographic evaluation to study the evolution of PH in BPD (10, 11).

Tissue Doppler imaging (TDI) is a newer echocardiographic technique that uses Doppler principles to measure the higher amplitude and lower velocity signals from myocardial tissue motion. TDI can be used to assess myocardial systolic and diastolic function by measuring long-axis ventricular motion. TDI has been validated in various pathological conditions in preterm infants, but longitudinal studies in BPD-PH are lacking (12, 13).

The primary aim of the study was to ascertain the value of serial right ventricular (RV) functional assessment in extremely premature infants when evaluating BPD-PH. We also aimed to evaluate the clinical predictors of BPD-PH and the optimal timing of echocardiographic screening in high-risk infants.

We hypothesize that serial TDI in premature infants will enable the early detection of pulmonary hypertension and increase the sensitivity of diagnosis when coupled with conventional echocardiography.

## Methodology

This is a single-center prospective observational study conducted in a large tertiary neonatal unit in Singapore. The study was initiated in September 2018 and the last participant was recruited in March 2021.

### Patient cohort

Infants born at <28 weeks of gestation were recruited into the study. We excluded infants born with major congenital anomalies. Each recruited infant had their first comprehensive echocardiogram involving both conventional and TDI at 7–10 days of life (scan 1) and 4-weekly scans (scan 2: 29.4 ± 1.17 weeks, scan 3: 33.8 ± 1 weeks, scan 4: 36.7 ± 0.5 weeks) thereafter until 36 weeks of corrected age. At a postmenstrual age of 36 weeks, the presence of BPD-PH was diagnosed using the conventional echocardiographic parameters. The infant was diagnosed with BPD-PH if there was a tricuspid regurgitation (TR) jet maximum velocity >2.8 m/s or a combination of left ventricular eccentricity index of more than 1.15 and the ratio of acceleration time to RV ejection time (ACT/RVET) <0.3 (14–16). The TDI markers were then compared between BPD-no PH and BPD-PH groups at each period.

Hemodynamically significant patent ductus arteriosus (Hs PDA) was defined as size >1.6 mm, left heart dilatation, reversal of flow in descending aorta, clinical signs of congestive heart failure, and needing treatment. The severity of BPD was defined as per the National Institute of Child Health and Human Development (NICHD) criteria (17). Severity of retinopathy of prematurity (ROP) was defined as per the International Classification of Retinopathy of Prematurity.

### Clinical data

Baseline clinical parameters were collected including maternal antenatal details and infants’ clinical and respiratory characteristics.

### Echocardiography

A Vivo S6 machine (GE Medical, Milwaukee, WI, USA) with 7 and 10 MHz probes was used for the cardiac scans. All study measurements were averaged for three to five cardiac cycles. The echocardiogram scans were performed by two senior cardiac technologists and all parameters were analyzed by a single study member (KG). The image acquisition and TDI measurements were standardized by a single senior pediatric cardiologist (SS). The initial standardization of scans and measurements was performed on six well preterm infants without any respiratory support before the study commencement. The calculated interclass correlation coefficient (ICC) between the study member and the cardiologist was 0.89. The ICC between cardiac technologists was 0.84. The unstable infants had their scans postponed to a later date. No infants received diuretics (>1 week) and pulmonary vasodilators during the study period.

We measured tricuspid jet velocity, TR systolic to diastolic ratio, ratio of pulmonary artery acceleration time to RV ejection time, RV dimensions on four-chamber, short axis, and long-axis views, LV eccentricity index (LVEI) (end diastole, end systole, average), and tricuspid and mitral E/A ratio as conventional echocardiographic parameters. Tissue Doppler images were obtained at the lateral mitral and tricuspid annulus (pulse wave on color-coded TDI). TDI measurements collected were isovolumetric contraction time, isovolumetric relaxation time, and ejection time. Other TDI parameters were S’, E’, A’, isovolumetric velocity, and E/E’ ratio. All the images were analyzed on site.

### Statistical analysis

Categorical variables were analyzed using the chi-square test, and continuous variables were analyzed with the Mann–Whitney *U*-test. We constructed a receiver operating characteristic (ROC) curve for echo parameters for statistically significant conventional and TDI parameters to predict the cut-off values in predicting BPD-PH. Clustered box plots were used to show the trend of significant cardiac parameters. The data were analyzed using SPSS statistics (Version 26; IBM Corp.) and a two-sided *p*-value <0.05 was considered statistically significant.

The study was approved by the centralized institute review board (CIRB 2018/2077).

## Results

**Figure 1:**
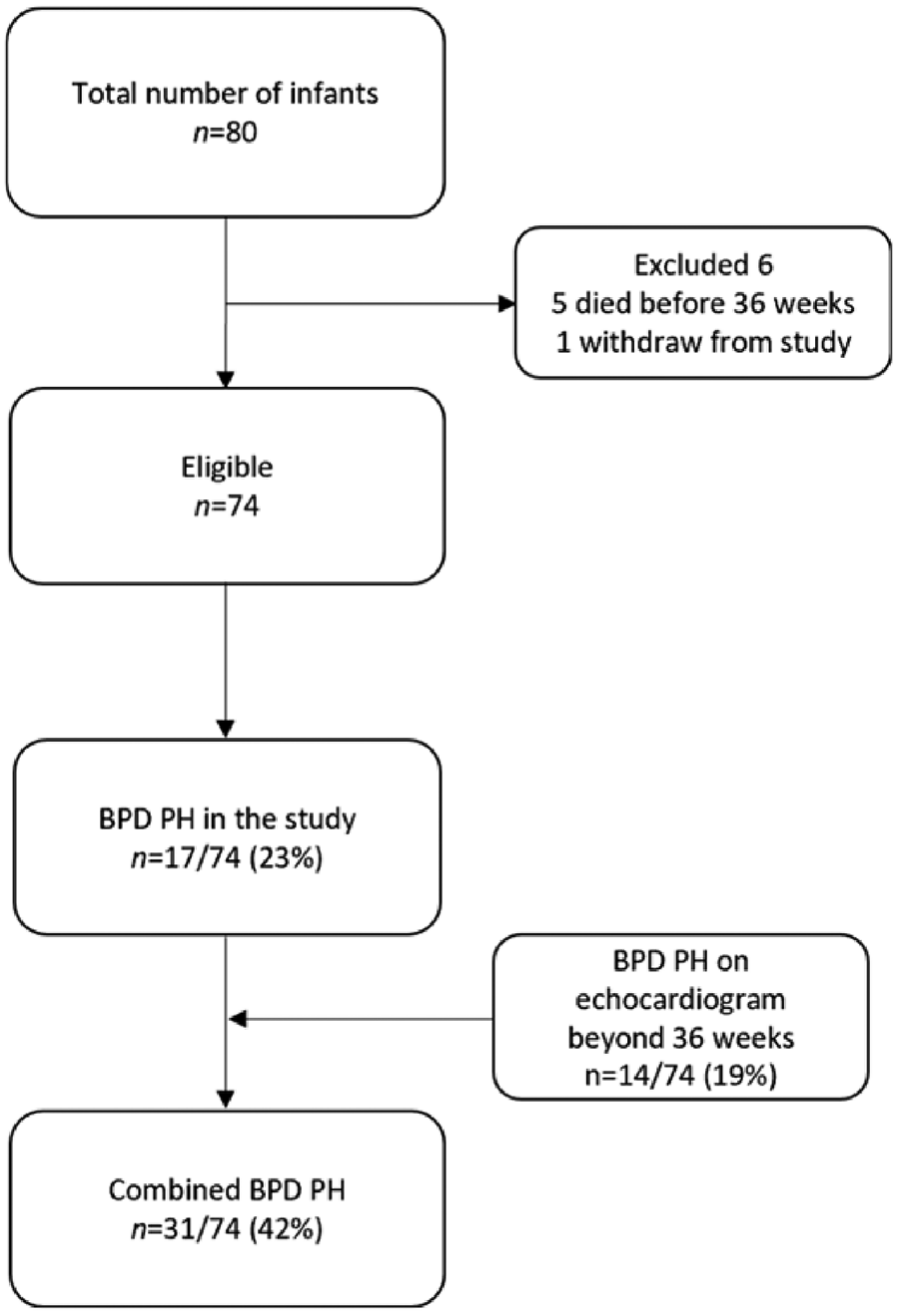
Study flow chart.

A total of 80 infants were recruited into the study, and 74 infants were included in the final analysis, as shown in Figure 1. Five infants died before the post-conceptual age of 36 weeks and one infant withdrew from the study. The incidence of BPD-PH was 23% in our cohort. An additional 14 infants showed PH on continued screening beyond 36 weeks of gestation (mean age of diagnosis 39.5 ± 6.42 weeks) making the total incidence of BPD-PH 41%.

The mean gestational age of the study population was 25.9 ± 1.1 weeks and the mean birth weight was 830 ± 174 g. Table 1 shows that 3 (18%) infants in the mild BPD group had evidence of PH, whereas the incidence was 82.4% (14/17) in moderate to severe BPD.

**Table 1:** Comparison of antenatal, neonatal, and other demographic characteristics between study.

	BPD without PH *n* = 48	BPD-PH (total)^a^ *n* = 31	*p*-value
Gestational age (weeks), mean ± SD	26.1 ± 1.2	25.7 ± 0.99	0.13
Birth weight (g), mean ± SD	869 ± 181	775 ± 149	0.02
Male, *n* (%)	26 (54.2)	14 (45.2)	0.43
Small for gestational age, *n* (%)	4 (8.3)	4 (12.9)	0.51
Vaginal delivery, *n* (%)	18 (37.5)	16 (51.6)	0.21
Antenatal risk factors			
PROM, *n* (%)	17 (35.4)	14 (45.2)	0.38
PIH, *n* (%)	4 (8.3)	3 (9.7)	0.83
APH, *n* (%)	14 (29.2)	9 (29)	0.99
Chorioamnionitis, *n* (%)	23 (52)	20 (66)	0.24
Neonatal characteristics			
Hemodynamically significant PDA, *n* (%)	24 (50)	25 (80.6)	0.006
Duration of CPAP/NIMV (days), mean ± SD	51.15 ± 28.6	60.7 ± 36.5	0.2
Duration of IPPV/HFOV (days), mean ± SD	13.77 ± 19.2	31.03 ± 40.4	0.01
Duration of oxygen therapy (days), mean ± SD	59.35 ± 52.1	96.16 ± 68.09	0.008
Fio2 at 28 days (%), mean ± SD	29.9 ± 12.21	33.68 ± 11.9	0.18
Fio2 at 36 weeks (%), mean ± SD	23.17 ± 5.8	30.07 ± 18.34	0.056
Total duration in hospital (days), mean ± SD	106.5 ± 37.9	133.8 ± 45.9	0.005
Home oxygen, *n* (%)	9 (20)	13 (43)	0.02
Mod/severe BPD, *n* (%)	32 (72)	26 (86)	0.06
Stage 2 or more ROP, *n* (%)	26 (54)	23 (74)	0.04
NEC stage 2 and above, *n* (%)	3 (6.3)	0	0.15
Culture positive LOS, *n* (%)	6 (12.5)	8 (25.8)	0.13
Postnatal steroid therapy, *n* (%)	14 (23.2)	18 (58.1)	0.01
Death, *n* (%)	2 (4.2)	6 (19.4)	0.03

PROM, premature rupture of membranes; PIH, pregnancy induced hypertension; APH, antepartum hemorrhage; PDA, patent ductus arteriosus; CPAP, continuous positive airway pressure; NIMV, non-invasive mandatory ventilation; IPPV, intermittent positive pressure ventilation; HFOV, high frequency oscillatory ventilation; BPD, bronchopulmonary dysplasia; ROP, retinopathy of prematurity; NEC (Bell's staging), necrotizing enterocolitis; LOS, late onset sepsis.

^a^ Total BPD-PH of the study cohort.

The BPD-PH group had a high incidence of Hs PDA compared to the BPD-no PH group (83% vs. 56%, *p* < 0.018), longer oxygen days (96.16 ± 68.09 vs. 59.35 ± 52.1, *p* < 0.008), invasive ventilation days (31.03 ± 40.4 vs. 13.77 ± 19.2, *p* < 0.01), and hospital stay (133.8 ± 45.9 days vs. 106.5 ± 37.9 days, *p* < 0.005).

In total, 13 infants in the PH group required home oxygen (13 vs. 9, *p* < 0.02). More infants in the BPD-PH group required postnatal steroid therapy (18 (58.1%) vs. 14 (29.2%), *p* < 0.01) and there was higher mortality in the BPD-PH group [6 (20%) vs. 2 (4.2%), *p* < 0.02]. There was no statistical difference in the incidence of severe BPD in both groups, though a higher number of infants in the severe BPD group were affected by PH. The rest of the study characteristics were not significant between the groups, as shown in Table 1.

Among the conventional echocardiographic parameters, the left ventricular eccentricity index (end-diastolic, end-systolic, and average) was higher in infants with BPD-PH (1.24 ± 0.1 vs. 0.97 ± 0.1, *p* < 0.001) and lower ACT/RVET ratio from 33 weeks (0.28 ± 0.05 vs. 0.24 ± 0.05, *p* < 0.05). These differences persisted at the 36-week scan. Infants with BPD-PH showed higher basal RV dimension from 33 weeks (11.8 ± 2.3 vs. 10.6 ± 1.75 mm, *p* < 0.04) and 36 weeks (13.6 ± 1.95 vs. 12.5 ± 1.8 mm, *p* < 0.03). The conventional echocardiographic features are summarized in Table 2.

**Table 2:** Serial conventional echocardiography between BPD-PH and no PH groups.

	Scan 1		Scan 2		Scan 3		Scan 4	
	BPD without PH	BPD-PH *n* = 17	BPD without PH	BPD-PH *n* = 17	BPD without PH	BPD-PH *n* = 17	BPD without PH	BPD-PH *n* = 17
SBP (mmHg)	59.56 ± 12.9	58.82 ± 10.82	66.67 ± 11.4	67.06 ± 11.5	80.19 ± 12	79.8 ± 9.2	82.7 ± 8	81.75 ± 8.7
DBP (mmHg)	33.75 ± 8.2	35.91 ± 9.1	38.48 ± 9.6	36.53 ± 8.7	44.32 ± 13.3	42.20 ± 9.4	45.2 ± 9.2	45.3 ± 11.2
LV EI end syst	1.03 ± 0.16	1.12 ± 0.18	1.03 ± 0.2	1.09 ± 0.2	**0.97 ** **± ** **0.15**	**1.1 ± 0.17** **	**0.99 **± **0.1**	**1.24 ± 0.1** ***
Basal RV (mm)	**8.74 **±** 1.53**	**10 **± 1.75**	8.99 ± 1.75	9.8 ± 1.9	10.6 ± 1.75	**11.8 ± 2.3** **	**12.5 **± **1.8**	**13.6 ± 1.95** *
Mid RV (mm)	7.21 ± 1.35	7.9 ± 1.01	7.85 ± 1.6	8.3 ± 1.66	9.17 ± 1.78	9.9 ± 1.88	**10.4 **± **1.7**	**11.6 ± 1.7** **
RV length (mm)	15.4 ± 1.99	16.9 ± 2.6**	17.02 ± 2.2	17.6 ± 2.6	20 ± 2.58	21.35 ± 2.64	22.5 ± 2.6	23.07 ± 2.8
PDA (mm)	2.09 ± 0.53	2.87 ± 0.93**	1.96 ± 0.52 *n* = 19	2.25 ± 0.86 *n* = 12	1.38 ± 0.3 *n* = 5	2.96 ± 1.53 *n* = 5	1.59 ± 0.95 *n* = 2	2.83 *n* = 1
TAPSE (mm)	6.5 ± 1.25	6.26 ± 0.56	7.33 ± 1.1	7 ± 1.2	8.7 ± 1.58	8.6 ± 1.3	9.9 ± 1.84	9.5 ± 1.63
RVIDd (mm) (Psax)	5.5 ± 0.82	5.4 ± 0.9	6.06 ± 1.2	6.67 ± 1.36	7.47 ± 1.24	7.7 ± 1.5	8.4 ± 1.41	8.73 ± 1.18
RVIDd (mm) (Plax)	5.4 ± 0.92	5.3 ± 0.67	**5.74 **± **1**	**6.6 ± 1.36** ***	7.15 ± 1.34	7.14 ± 1.7	7.6 ± 1.21	**8.6 ± 1.2** **
TR (m/s)	2.22 ± 0.37	2.53 ± 0.36	**2.2 **± **0.36**	**3 ± .54** ***	2.3 ± 0.2	2.45 ± 0.64	2.32 ± 0.2	2.21 ± 1.2
PV VTI (cm)	12.12 ± 4.5	10.43 ± 3.23	12.6 ± 3.8	12.8 ± 4.9	12.9 ± 2.7	14.1 ± 2.46	13.2 ± 2.4	**14.6 ± 2.5** *
TR/VTI	19.48 ± 4.28	25.56 ± 10.1	18.18 ± 4.6	23.5 ± 5.11**	18.78 ± 3.24	16.8 ± 4.9	18.76 ± 3.8	15.4 ± 8.8
TR SD/DD	1.28 ± 0.48	1.9 ± 0.49**	1.24 ± 0.26	1.64 ± .57	1.56 ± 0.28	1.66 ± 0.7	1.52 ± 0.4	1.66 ± 0.4
ACT (ms)	62.59 ± 19.81	50 ± 20.4	60.4 ± 18.3	61.54 ± 23.7	**55.45 **± **13.5**	**48.06 ± 8.1** *	54.6 ± 13.3	49.1 ± 7.9
RVET (ms)	181.39 ± 32.08	175 ± 41.17	194.6 ± 32.5	186.6 ± 32	196.25 ± 20.3	202.8 ± 47	193.9 ± 22.4	202.4 ± 22.8
ACT/RVET	0.34 ± 0.1	0.28 ± 0.09	0.31 ± 0.1	0.33 ± 0.12	**0.28 **± **0.05**	**0.24 ± 0.05** *	**0.28 **± **0.06**	**0.24 ± 0.03** **

SBP, systolic blood pressure; DBP, diastolic blood pressure; LV, left ventricle; EI, eccentricity index; RV, right ventricle; PDA, patent ductus arteriosus; TAPSE, tricuspid annular plane systolic excursion; RVIDd, right ventricle internal diameter; Psax, parasternal short axis; Plax, parasternal long axis; TRVmax, tricuspid regurgitation velocity; PV VTI, pulmonary valve velocity time integral; SD/DD, systolic to diastolic ratio; PA ACT, pulmonary arterial acceleration time; RVET, right ventricular ejection time; ACT/RVET, acceleration time to RV ejection ratio; TV, tricuspid valve.

All the values expressed in mean ± SD. Statistically significant values are in bold.

* *p* < 0.05.

** *p* < 0.01.

*** *p* < 0.001.

The RV TDI parameters isovolumetric contraction time (IVCT), isovolumetric relaxation time (IVRT), and RV myocardial performance index (MPI) showed significant differences between the groups. At the 33-week scan, the BPD-PH group showed prolonged IVCT (27.84 ± 5.5 vs. 22.77 ± 4 ms, *p* < 0.001), IVRT (40.3 ± 7.1 vs. 34.9 ± 5.3 ms *p* < 0.003), and abnormal MPI (0.39 ± 0.05 vs. 0.32 ± 0.03, *p* < 0.001). The ejection time was shorter in infants with BPD-PH at the 36-week scan (168 ± 12.3 vs. 183 ± 18.5 ms, *p* < 0.004). These differences persisted at 36 weeks, as shown in Table 3. The TDI parameters showed a similar trend when adjusted for gestational age at birth.

**Table 3:** Serial RV TDI functional assessment between study groups.

RV TDI	Scan 1		Scan 2		Scan 3		Scan 4	
	BPD without PH	BPD-PH	BPD	BPD-PH	BPD	BPD-PH	BPD	BPD-PH
RV E’ (cm/s)	6.4 ± 2.6	6.36 ± 2.7	6.34 ± 1.6	7.07 ± 1.7	7.4 ± 1.1	7.8 ± 1.8	9.2 ± 2.5	9.5 ± 1.6
RV E/E’	7.5 ± 2.7	6.4 ± 1.5	7.14 ± 1.7	6.3 ± 1.98	6.14 ± 1.7	7.48 ± 1.74	6.4 ± 1.8	5.9 ± 1.6
RV A’ (cm/s)	9.7 ± 1.5	9.9 ± 1.7	10.7 ± 1.9	9.5 ± 2.84	11.3 ± 1.8	11.6 ± 1.3	11.8 ± 3.5	12.1 ± 2.1
RV S’ (cm/s)	6.5 ± 1.37	6.8 ± 1.34	7.64 ± 1.41	7.55 ± 1.4	8.3 ± 1.6	8.5 ± 1.28	9.35 ± 1.9	9.8 ± 2.1
RV-IVV (cm/s)	5.3 ± 1.4	4.89 ± 1.6	6.2 ± 1.79	5.8 ± 21	6.8 ± 2.1	5.7 ± 1.42	7.2 ± 2.2	7.05 ± 2.2
IVCT (ms)	24.13 ± 4.1	26.24 ± 8.6	23.1 ± 4.2	25.4 ± 7.43	**22.77 ** **± ** **4.04**	**28 ± 5.5** ***	**23.73 **± **3.5**	**25.8 ± 4.6** *
IVRT (ms)	40.06 ± 7.6	37.89 ± 5.7	38.7 ± 8.7	38.2 ± 6.1	**34.9 **± **5.3**	**40 ± 7.1** ***	**33.6 **± **5.7**	**54 ± 8.4** ***
ET (ms)	165.91 ± 12	170 ± 10.2	167.5 ± 13.7	164.5 ± 12.6	**177 **± **13.54**	**171 **± **17.22**	**183 **± **18.5**	**168 ± 12.3** **
RV MPI	0.39 ± 0.06	0.37 ± 0.05	0.36 ± 0.05	0.39 ± 0.05	**0.32 **± **0.03**	**0.39 ± 0.05** ***	**0.31 **± **0.03**	**0.47 ± 0.05** ***

RV, right ventricle, IVV, isovolumetric volume velocity; IVCT, isovolumetric contraction time; IVRT, isovolumetric relaxation time; ET, ejection time; MPI, myocardial performance index.

Statistically significant values are in bold.

* *p* < 0.05.

** *p* < 0.01.

*** *p* < 0.001.

**Figure 2:**
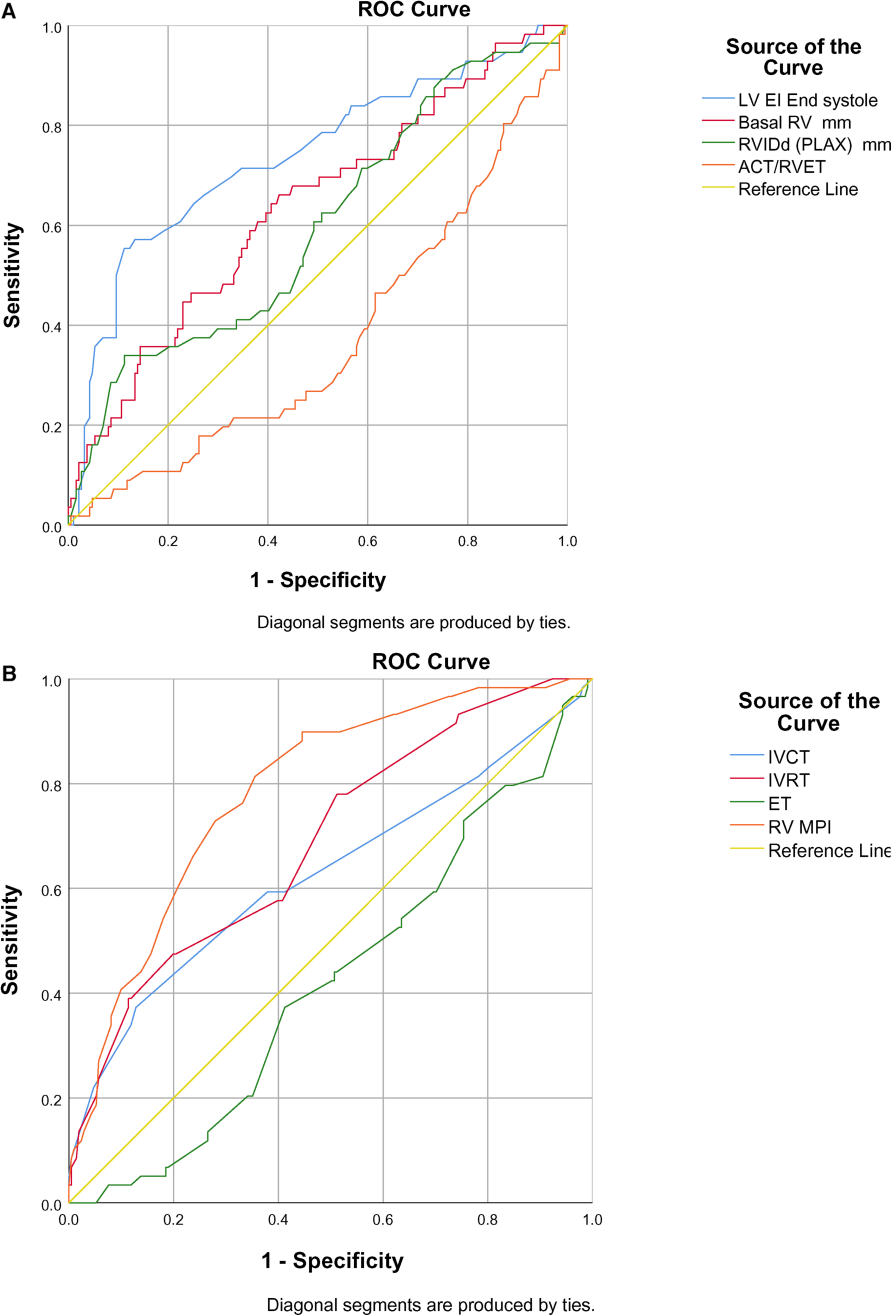
(**A**) ROC curve of conventional echocardiographic parameters in predicting BPD-PH. (**B**) ROC curve of TDI parameters in predicting BPD-PH.

The ROC curve showed end-systolic LV EI >1.16 had a sensitivity of 94% and specificity of 98% when diagnosing BPD-PH [area under the curve (AUC) 0.734, *p* < 0.001]. The RV internal diameter of >8.46 mm at 36 weeks had a sensitivity of 76% and specificity of 75% when diagnosing BPD-PH. The ACT/RVET ratio of 0.25 had a low sensitivity (41%) and specificity (40%) when diagnosing PH (AUC 0.304, *p* < 0.016), as shown in Figure 2A.

**Figure 3:**
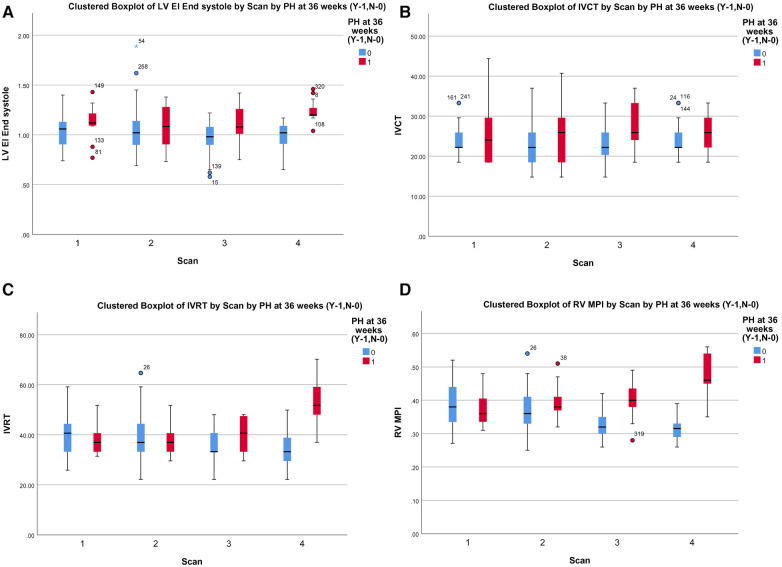
(**A**) Cluster box plot of LV EI. (**B**) Cluster box plot of IVCT. (**C**) Cluster box plot of IVRT. (**D**) Cluster box plot of MPI.

Among the TDI parameters, the RV MPI had better predictability (AUC 0.786, *p* < 0.001) followed by IVRT (AUC 0.688, *p* < 0.001) and IVCT (AUC 0.628, *p* < 0.003), as shown in Figure 2B. The RV MPI of 0.38 had a sensitivity of 66% and specificity of 76%, the IVCT of >25 ms had a sensitivity of 59% and specificity of 62%, and the IVRT >40 ms had a sensitivity and specificity of 57% and 60%, respectively, when diagnosing BPD-PH. The clustered box plots for echo parameter (LV EI, IVCT, IVRT, RV MPI) trends at different time points is shown in Figures 3A–D.

## Discussion

The incidence of PH in our study was 23% (17/74) at the 36-week scan. The total incidence of PH increased to 41% on echocardiogram screening beyond 36 weeks of post-conceptual age. Our results are similar to those of previously reported prospective studies (7, 18, 19): 3 (18%) infants with mild BPD had PH, which is higher than in previously published studies except in a study by Sun et al., which showed a 33% incidence of mild BPD (6, 20). The abnormal fetal vascular modeling might be the reason for elevated vascular resistance in this group (21). This highlights the importance of screening infants with all grades of BPD for PH.

In our study, BPD-PH had a higher incidence of hemodynamically significant PDA. PDA as a significant predictor of BPD-PH has been described by others (5, 7, 22). The prolonged exposure to PDA results in structural and functional changes in developing pulmonary arterioles resulting in elevated pulmonary arterial pressures (23). Our study demonstrated the worst outcome of respiratory morbidities in terms of the need for prolonged respiratory support, oxygen therapy, hospital stay, and higher mortality in the BPD-PH cohort (19, 20, 24, 25).

The LV EI is an objective measure of septal configuration at a modified short-axis view (Figure 1, echo) and is a useful marker of RV systolic hypertension (26). In our study, LVEI, ACT/RVET, and basal RV dimensions were statistically significantly different from 33 weeks onwards. Abraham and Weismann showed LV EI >1.15 correlates with systolic septal flattening and a ratio >1.3 was associated with RV functional impairment (14). The utility of age-dependent LV EI and cut-off values in predicting PH has also been validated by Schweintzger et al. (27). Our study showed an end-systolic LV EI >1.16 had high sensitivity and specificity in diagnosing BPD-PH.

The ACT/RVET has been correlated with catheter-measured pulmonary pressure (28). The lower ratio <0.31 reliably detected elevated pulmonary vascular resistance with high sensitivity and specificity in older children (9, 29). In our study, the BPD-PH group had a lower ratio (<25) but had lower sensitivity and specificity. Nonetheless, this is a simple cardiac parameter that can be used in conjunction with other cardiac parameters in the diagnosis of BPD-PH. The ACT time was not significant between the two cohorts in our study. ACT is known to be shorter in neonates due to the faster heart rate and is well validated in older children with PH (30).

TR Vmax >2.8 m/s, which has high sensitivity in the diagnosis of PH, was present in only 16 (43%) infants. Other TR-derived measures, such as the SD/DD ratio and TR/VTI ratio, were not significant among the study groups.

Tricuspid annular plane systolic excursion (TAPSE) is one of the widely studied parameters to measure longitudinal RV lateral wall performance, which correlates with RV ejection fraction (31). TAPSE linearly increases with gestational age in preterm neonates and was significantly lower in patients with PH (32, 33). We did not find a difference in TAPSE between the groups, though the PH group had lower values. A recent meta-analysis showed Fractional area change (FAC) may have a better correlation with RV systolic function compared to TAPSE (34). TDI has been shown to correlate with invasive pulmonary hemodynamics in pediatric PH (35). IVCT, measured by the end of the a’ wave to the beginning of the S’ wave, has shown a good correlation with ventricular systolic dysfunction in various neonatal conditions (36). IVRT is measured by the end of the S’ wave to the beginning of e’ wave, is a marker of diastolic dysfunction. It is inversely correlated with the cardiac index (37). It has been well validated in pulmonary artery systolic pressure in adult patients (38).

The utility of TDI parameters, such as MPI, E/e’ ratio, RV peak systolic, isovolumic velocities, IVCT, and IVRT, has been well documented in infants with BPD-PH (39–42). Our study showed higher IVCT, IVRT, and higher MPI in the BPD-PH cohort. These changes were consistent from 33 weeks.

**Figure 4:**
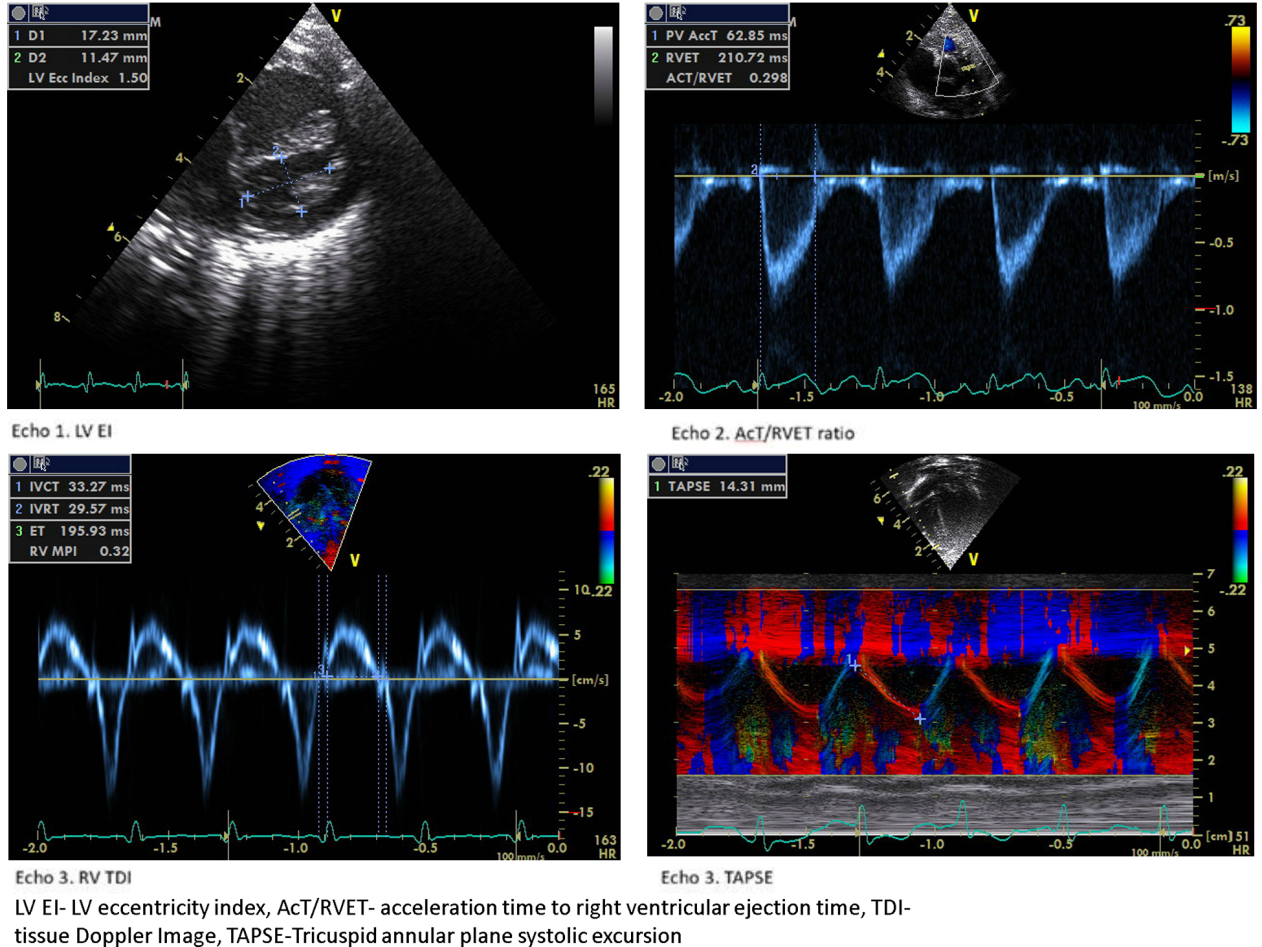
Echocardiogram images of the study population.

We observed a decreased RV E’ and E/E’, a marker of diastolic dysfunction in the BPD-PH group, but the difference was not statistically significant. The myocardial performance (Tei) index is a measure of global ventricular function (43). Patel et al. showed significantly higher RV MPI in infants with PH but a poor correlation with pulmonary artery pressure measured by TR (44). We found that RV MPI had a high predictive value followed by IVRT and IVCT. To the best of our knowledge, this is the first study to show the serial RV functional assessment in the evolution of BPD-PH. Figure 4 shows the sample 2D echocardiogram images of various conventional and TDI images from the study.

### Limitations

The present study has some limitations. The smaller sample size is one of the limitations. Our study lacks the direct comparison of TDI with gold standard cardiac catheterization in the diagnosis of PHT, though we attempted to increase the robustness of diagnosis by incorporating multiple echo parameters.

## Conclusions

Serial TDI along with conventional echocardiogram increases the diagnostic accuracy of BPD-PH. The TDI parameters MPI and IVRT are sensitive in recognizing early RV stress and should be incorporated in routine screening. LV end-systolic EI, ACT/RVET ratio, and RV dimensions can be incorporated into the PH screening protocol in the absence of TR. We recommend initiating PH screening from 32 to 34 weeks and continuing to screen beyond 36 weeks in high-risk infants.

## Data Availability

The original contributions presented in the study are included in the article/Supplementary Material, further inquiries can be directed to the corresponding author.
